# Imatinibium dipicrate

**DOI:** 10.1107/S1600536810000577

**Published:** 2010-01-20

**Authors:** Jerry P. Jasinski, Ray J. Butcher, Q. N. M. Hakim Al-Arique, H. S. Yathirajan, B. Narayana

**Affiliations:** a Department of Chemistry, Keene State College, 229 Main Street, Keene, NH 03435-2001, USA; b Department of Chemistry, Howard University, 525 College Street NW, Washington, DC 20059, USA; c Department of Studies in Chemistry, University of Mysore, Manasagangotri, Mysore 570 006, India; d Department of Studies in Chemistry, Mangalore University, Mangalagangotri 574 199, India

## Abstract

In the crystal structure of imatinibium dipicrate [systematic name: 1-methyl-4-(4-{4-methyl-3-[4-(3-pyrid­yl)pyrimidin-2-yl­amino]­anilinocarbon­yl}benz­yl)piperazine-1,4-diium dipicrate], C_29_H_33_N_7_O^2+^·2C_6_H_2_N_3_O_7_^−^, the imatinibium cation is proton­ated at both of the pyrimidine N atoms. Each of the two picrate anions inter­acts with the diprotonated cation through bifurcated N—H⋯O hydrogen bonds forming *R*_1_^2^(6) ring motifs. Also, an *R*_2_^2^(24) graph set is formed between the benzamidium –NH– group and the 4-pyridyl N atom inter­acting through N—H⋯N hydrogen-bond inter­actions. Additional weak C—H⋯*Cg* π-ring and π–π inter­molecular inter­actions are observed which also influence crystal packing.

## Related literature

For related structures, see: Bindya *et al.* (2007 ▶); Harrison, Bindya *et al.* (2007 ▶); Harrison, Sreevidya *et al.* (2007 ▶); Jasinski *et al.* (2009*a* ▶,*b* ▶); Swamy *et al.* (2007 ▶); Szumma *et al.* (2000 ▶); Yathirajan *et al.* (2007*a* ▶,*b* ▶). For a rationally developed anticancer drug, see: Capdeville *et al.* (2002 ▶). For its use in chronic myeloid leukaemia, see: Moen *et al.* (2007 ▶). For puckering parameters, see: Cremer & Pople (1975 ▶).
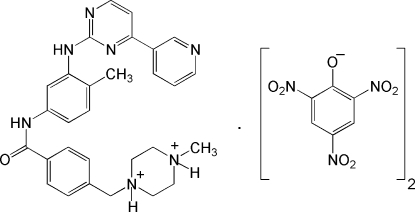


## Experimental

### 

#### Crystal data

C_29_H_33_N_7_O^2+^·2C_6_H_2_N_3_O_7_^−^
*M* *_r_* = 951.84
Triclinic, 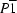

*a* = 8.560 (1) Å
*b* = 10.734 (1) Å
*c* = 23.060 (1) Å
α = 96.74 (3)°
β = 92.69 (2)°
γ = 101.46 (7)°
*V* = 2056.9 (6) Å^3^
*Z* = 2
Cu *K*α radiation
μ = 1.02 mm^−1^
*T* = 110 K
0.45 × 0.39 × 0.24 mm

#### Data collection

Oxford Diffraction Xcalibur diffractometer with a Ruby (Gemini Cu) detector
Absorption correction: multi-scan (*CrysAlis RED*; Oxford Diffraction, 2007 ▶) *T*_min_ = 0.596, *T*_max_ = 0.782
15890 measured reflections
8082 independent reflections
6946 reflections with *I* > 2σ(*I*)
*R*_int_ = 0.023

#### Refinement

*R*[*F* ^2^ > 2σ(*F* ^2^)] = 0.059
*wR*(*F* ^2^) = 0.158
*S* = 1.06
8082 reflections
640 parameters
H atoms treated by a mixture of independent and constrained refinement
Δρ_max_ = 0.50 e Å^−3^
Δρ_min_ = −0.27 e Å^−3^

### 

Data collection: *CrysAlis PRO* (Oxford Diffraction, 2007 ▶); cell refinement: *CrysAlis RED* (Oxford Diffraction, 2007 ▶); data reduction: *CrysAlis RED*; program(s) used to solve structure: *SHELXS97* (Sheldrick, 2008 ▶); program(s) used to refine structure: *SHELXL97* (Sheldrick, 2008 ▶); molecular graphics: *SHELXTL* (Sheldrick, 2008 ▶) and *Mercury* (Macrae *et al.*, 2006 ▶); software used to prepare material for publication: *SHELXTL*, *enCIFer* (Allen *et al.*, 2004 ▶) and *PLATON* (Spek, 2009 ▶).

## Supplementary Material

Crystal structure: contains datablocks global, I. DOI: 10.1107/S1600536810000577/bt5129sup1.cif

Structure factors: contains datablocks I. DOI: 10.1107/S1600536810000577/bt5129Isup2.hkl

Additional supplementary materials:  crystallographic information; 3D view; checkCIF report

## Figures and Tables

**Table 1:** Hydrogen-bond geometry (Å, °)

*D*—H⋯*A*	*D*—H	H⋯*A*	*D*⋯*A*	*D*—H⋯*A*
N1—H1⋯O1*B*	0.85 (3)	1.85 (3)	2.658 (3)	157 (3)
N1—H1⋯O62*B*	0.85 (3)	2.35 (3)	2.890 (3)	122 (2)
N2—H2⋯O1*A*	0.89 (4)	1.85 (4)	2.678 (3)	154 (3)
N2—H2⋯O62*A*	0.89 (4)	2.41 (4)	3.009 (3)	125 (3)
N14—H14⋯N31^i^	0.85 (3)	2.23 (3)	3.069 (3)	171 (3)
C5—H5*B*⋯O41*A*^ii^	0.98	2.48	3.258 (4)	136
C4—H4*B*⋯O42*B*^iii^	0.99	2.33	3.199 (3)	146
C3—H3*A*⋯O61*B*^iv^	0.99	2.57	3.199 (3)	121
C3—H3*B*⋯O1*B*	0.99	2.34	3.072 (3)	130
C12—H12*A*⋯O42*B*^iii^	0.95	2.63	3.423 (3)	142
C19—H19*A*⋯O61*B*^v^	0.98	2.50	3.435 (4)	159
C19—H19*A*⋯N6*B*^v^	0.98	2.65	3.541 (4)	152

Symmetry codes: (i) 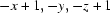; (ii) 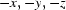; (iii) 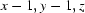; (iv) 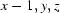; (v) 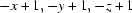.

**Table 2:** π-Ring hydrogen-bond geometry (Å, °) for (I)

*D*—H⋯*A*	*D*—H	H⋯*A*	*D*⋯*A*	*D*—H⋯*A*
C33—H33*A*⋯*Cg*5^vi^	0.95	2.90	3.545 (8)	127

Symmetry code: (vi) *x* + 1, *y*, *z*. *Cg5* is the centroid of the C15–C21 ring.

**Table 3:** π–π stacking geometry (Å) for (I)

*Cg*2⋯*Cg*7^v^	3.740 (4)
*Cg*3⋯*Cg*3^v^	3.496 (7)
*Cg*6⋯*Cg*6^vii^	3.396 (0)

Symmetry codes: (v) −*x* + 1, −*y* + 1, −*z* + 1; (vii) −*x* + 2, −*y* + 2, −*z*. *Cg*2, *Cg*3, *Cg*6 and *Cg*7 are the centroids of the C25–C27/N28/C23/N4, C32–C34/C29//C30/N31, C1*A*–C6*A* and C1*B*–C6*B* rings, respectively.

**Fig. 1.**
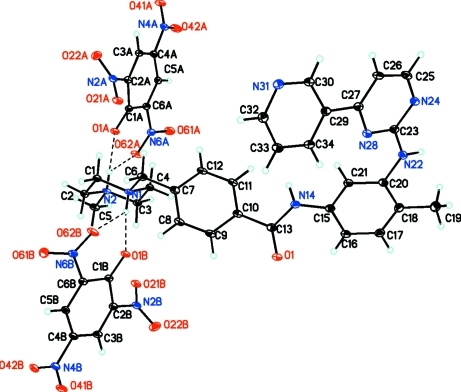
Molecular structure of the title compound, C29H33N7O2+ (C6H2N3O7-)2, showing the cation-dianion unit that comprises the asymmetric unit, the atom labeling scheme and 30% probability displacement ellipsoids. Picrate anions A & B are labeled accordingly.

**Fig. 2.**
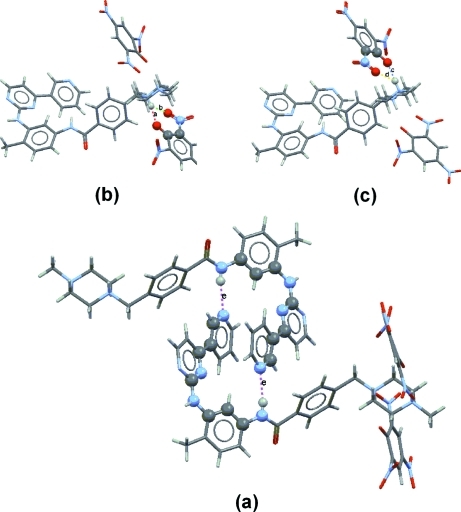
Diagrams of the (a) R22(24) ···ee···, (b) R21(6) ···ab··· and (c) R21(6) ···cd··· graph-set motifs in the cation (a) and anions (b, c) of the title compound, (I).

**Fig. 3.**
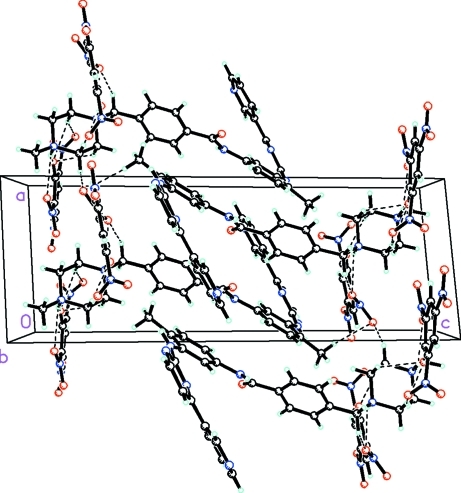
Packing diagram of the title compound, (I), viewed down the b axis. Dashed lines indicate intermolecular N—H···O, N—H···N & C—H···O hydrogen bond interactions which produces a two-dimensional network arranged along the (101) plane of the unit cell.

## supplementary crystallographic information

### Comment

Imatinib, marketed as a cancer drug by Novartix, [Gleevec(USA), Glivec(Europe/Australia)] (systematic name: 4-(4-methyl-piperazin-1-yl-methyl-*N*-[4-methyl-3-(4-pyridin-3-ylpyrimidin-2-yl-amino)-phenyl]-benzamide) is a synthetic tyrosine kinase inhibitor used in treating chronic myelogenous leukemia (CML), gastrointestinal stromal tumours (GISTs) and a number of other malignancies. It is a 2-phenylaminopyrimidine derivative and is the first member of a new class of agents that act by inhibiting particular tyrpsine kinase enzymes, instead of non-specifically inhibiting rapidly dividing cells. Reviews on the use of imatinib in chronic myeloid leukaemia (Moen *et al.*, 2007) and on the rationally developed targeted anticancer drug have been published (Capdeville *et al.*, 2002). Picrates form charge-transfer complexes with organic compounds, function as acceptors in the formation of π- stacking complexes with aromatic biomolecules and as an acidic ligand forming salts with polar biomolecules. In this context, the crystal and molecular structures of related compounds include amitriptylinium picrate (Bindya *et al.*, 2007), mepazinium picrate (Yathirajan *et al.*, 2007a), trifluperazinium dipicrate (Yathirajan *et al.*, 2007b), imipraminium picrate (Harrison, Bindya *et al.*, 2007), nevirapiniumpicrate (Harrison, Sreevidya *et al.*, 2007), desipraminium picrate (Swamy *et al.*, 2007) and propiverinium picrate (Jasinski *et al.*, 2009a) have been reported. In view of the importance of imatinib and to study the hydrogen bonding patterns in the title compound, (I), C_29_H_33_N_7_O^2+^ (C_6_H_2_N_3_O_7_^-^)_2_, a dipicrate salt of Imatinib, a crystal structure is reported.

The imatinibium cation contains a doubly charged methyl piperazine group bonded at the 4 position of a *p*-methyl benzamide group and a 2-phenylaminopyrimidine(pyridine) derivative bonded to the amino end. The 6-membered methyl piperazine group adopts a slightly distorted chair conformation (Cremer & Pople, 1975) with puckering parameters Q, θ and φ of 0.572 (5) Å, 176.1 (5)° and 168.174 (3)°, respectively (Fig. 1). For an ideal chair θ has a value of 0 or 180°. An *R*_2_^2^(24) graph-set motif is formed between the benzamidium –NH– group and the 4-pyridiyl N atom interacting through a N–H···N hydrogen bond interaction (Fig.2a). The dihedral angle between the mean plane of the benzyl ring in the benzamide group and the mean planes of the piperazine, amino phenyl, pyrimidine and pyridine groups are 81.1 (7)°, 50.8 (5)°, 57.1 (7)° and 46.1 (4)°, respectively. The mean planes of the pyrimidine and pyridine rings are twisted by 11.1 (9)°. The dihedral angles between mean planes of the aminobenzyl group and the pyrimidine and pyridine groups are 30.7 (3)° and 32.3 (2)°, while the dihedral angles between the mean planes of the piperizine group and the aminobenzyl, pyrimidine and pyridine groups are 48.3 (9)°, 59.2 (1)° and 69.3 (9)°, respectively. The two picrate anions, labeled A and B, each interact with the diprotonated cation through bifurcated N–H···O hydrogen bonds forming an *R*_2_^1^(6) ring-motif creating an ···ab··· and ···cd··· array of hydrogen bonding patterns (Fig.2 b,c). The mean plane of the two *o*-NO_2_ groups in the two picrate anions are twisted by 16.1 (9)° and 39.1 (9)° in the A-ring and 27.1 (5)° and 47.4 (1)° in the B-ring with respect to the mean planes of the 6-membered benzene rings. The difference in the twist angles of the mean planes of the two *o*-NO_2_ groups in each picrate anion can be attributed to an intermolecular "side" hydrogen bond interaction (Szumma *et al.*, 2000) between the N1 and N2 atoms of the cation piperizine group with a two-centered hydrogen bond to the singly bonded oxygen atom (O1A & O1B) and to one oxygen atom of an adjacent *o*-NO_2_ group (O62A & O62B), respectively, [N1—H1···O1B & N1—H1···O62B and N2—H2···O1A & N2—H2···O62A, see Table 1, Fig.1]. The difference in angles between the mean planes of the *o*-O61A—N6A—O62A (16.1 (9)°) and *o*-O21*a*—N2a—O22A (39.1 (9)°) groups with the mean plane of the benzene ring in picrate A (23°) and those of the *o*-O21B—N2B—O22B (47.4 (1)°) and *o*-O61B—N6B—O62B (27.1 (5)°) with the mean plane of the benzene ringin picrate B (16.2 (3)°) are a direct result of the N2—H2···O62A and N1—H1···O62B hydrogen bonds. The *p*-NO_2_ groups in both picrate anions are essentially in the plane of the ring (torsion angles C5A—C4A—N4A—O41A = 179.9 (2)°; C5B—C4B—N4B—O41B = 176.1 (2)°). Crystal packing is also influenced by N—H···N hydrogen bond interactions between the benzamide and pyridine groups (N14—H14···N31), intermediate C—H···O hydrogen bond interactions (C5—H5B···O41A, C4—H4···H42B & C3—H3A···O61B) between the piperizine group and *o*-NO_2_ & *p*-NO_2_ groups of picrates A & B and weak C—H···O hydrogen bond interactions involving the benzamide, phenyl, *o*-NO_2_ and*p*-NO_2_ groups (C12—H12A···O42B, C19—H19···O61B & C19—H19A···N6B;Table 1) which produces a two-dimensional network arranged along the (101)plane of the unit cell (Fig.3). In addition there are weak C—H···π   (Table 2) and weak π-π intermolecular interactions (Table 3) similar to that observed in 3-(2-Chloroethyl)-2-methyl-4*H*-pyrido[1,2-*a*] pyrimidinium-4-one picrate (Jasinski *et al.*, 2009b).

### Experimental

The title compound was synthesized by mixing an aqueous solution (10 ml) of picric acid (0.92 g, 2 mmol) and *N*-(4-methyl-3-(4-(pyridin-3-yl)pyrimidin-2-ylamino)phenyl)-4-((4-methyl piperazin-1-yl)methyl)benzamide (1.18 g, 2 mmol) in methanolic aqueous solution (10 ml) and the resulting solution was stirred well at 313 K. The formation of a yellow precipitate of the charge transfer complex was noticed almost instantaneously. The formed complex was filtered off, washed with distilled water and dried *in vacuo* over CaCl_2_. The purity of the synthesized compound was improved by a successive recrystallization process with methanol (yield: 76.2%). The crystals for X-ray studies were grown from slow evaporation of a methanol solution. The melting range was found to be 490–493 K.

### Refinement

All of the H atoms were placed in their calculated positions and then refined using the riding model with N—H = 0.85–0.89, C—H = 0.95–0.99 Å, and with *U*_iso_(H) = 1.15–1.51*U*_eq_(C,*N*).

### Crystal data

**Table:**

C_29_H_33_N_7_O^2+^·2C_6_H_2_N_3_O_7_^−^	*Z* = 2
*M**_r_* = 951.84	*F*(000) = 988
Triclinic, *P*1	*D*_x_ = 1.537 Mg m^−^^3^
Hall symbol: -P 1	Cu *K*α radiation, λ = 1.54178 Å
*a* = 8.560 (1) Å	Cell parameters from 9389 reflections
*b* = 10.734 (1) Å	θ = 4.2–74.0°
*c* = 23.060 (1) Å	µ = 1.02 mm^−^^1^
α = 96.74 (3)°	*T* = 110 K
β = 92.69 (2)°	Chunk, pale yellow
γ = 101.46 (7)°	0.45 × 0.39 × 0.24 mm
*V* = 2056.9 (6) Å^3^	

### Data collection

**Table:**

Oxford Diffraction Xcalibur diffractometer with a Ruby (Gemini Cu) detector	8082 independent reflections
Radiation source: Enhance (Cu) X-ray Source	6946 reflections with *I* > 2σ(*I*)
graphite	*R*_int_ = 0.023
Detector resolution: 10.5081 pixels mm^-1^	θ_max_ = 74.1°, θ_min_ = 4.2°
ω scans	*h* = −10→8
Absorption correction: multi-scan (*CrysAlis RED*; Oxford Diffraction, 2007)	*k* = −13→13
*T*_min_ = 0.596, *T*_max_ = 0.782	*l* = −28→28
15890 measured reflections	

### Refinement

**Table:**

Refinement on *F*^2^	Primary atom site location: structure-invariant direct methods
Least-squares matrix: full	Secondary atom site location: difference Fourier map
*R*[*F*^2^ > 2σ(*F*^2^)] = 0.059	Hydrogen site location: inferred from neighbouring sites
*wR*(*F*^2^) = 0.158	H atoms treated by a mixture of independent and constrained refinement
*S* = 1.06	*w* = 1/[σ^2^(*F*_o_^2^) + (0.0615*P*)^2^ + 3.3059*P*] where *P* = (*F*_o_^2^ + 2*F*_c_^2^)/3
8082 reflections	(Δ/σ)_max_ = 0.001
640 parameters	Δρ_max_ = 0.50 e Å^−^^3^
0 restraints	Δρ_min_ = −0.27 e Å^−^^3^

### Special details

**Table:**

Geometry. All e.s.d.'s (except the e.s.d. in the dihedral angle between two l.s. planes) are estimated using the full covariance matrix. The cell e.s.d.'s are taken into account individually in the estimation of e.s.d.'s in distances, angles and torsion angles; correlations between e.s.d.'s in cell parameters are only used when they are defined by crystal symmetry. An approximate (isotropic) treatment of cell e.s.d.'s is used for estimating e.s.d.'s involving l.s. planes.
Refinement. Refinement of *F*^2^ against ALL reflections. The weighted *R*-factor *wR* and goodness of fit *S* are based on *F*^2^, conventional *R*-factors *R* are based on *F*, with *F* set to zero for negative *F*^2^. The threshold expression of *F*^2^ > σ(*F*^2^) is used only for calculating *R*-factors(gt) *etc*. and is not relevant to the choice of reflections for refinement. *R*-factors based on *F*^2^ are statistically about twice as large as those based on *F*, and *R*- factors based on ALL data will be even larger.

### Fractional atomic coordinates and isotropic or equivalent isotropic displacement parameters (Å^2^)

**Table:**

	*x*	*y*	*z*	*U*_iso_*/*U*_eq_	
O1	0.3153 (2)	0.58462 (17)	0.49702 (9)	0.0356 (4)	
N1	0.4213 (2)	0.4360 (2)	0.19545 (10)	0.0242 (4)	
H1	0.465 (4)	0.514 (3)	0.2058 (13)	0.028 (8)*	
N2	0.2325 (3)	0.4773 (2)	0.09313 (10)	0.0259 (5)	
H2	0.182 (4)	0.397 (4)	0.0809 (15)	0.044 (9)*	
C1	0.4665 (3)	0.4017 (2)	0.13475 (12)	0.0274 (5)	
H1A	0.4187	0.3106	0.1211	0.033*	
H1B	0.5841	0.4127	0.1349	0.033*	
C2	0.4094 (3)	0.4851 (2)	0.09331 (12)	0.0291 (5)	
H2A	0.4655	0.5752	0.1049	0.035*	
H2B	0.4371	0.4581	0.0532	0.035*	
C3	0.1853 (3)	0.5056 (2)	0.15391 (11)	0.0264 (5)	
H3A	0.0675	0.4925	0.1534	0.032*	
H3B	0.2307	0.5965	0.1689	0.032*	
C4	0.2433 (3)	0.4209 (2)	0.19414 (11)	0.0255 (5)	
H4A	0.2118	0.4431	0.2342	0.031*	
H4B	0.1922	0.3303	0.1807	0.031*	
C5	0.1813 (4)	0.5660 (3)	0.05447 (13)	0.0342 (6)	
H5A	0.0661	0.5612	0.0558	0.051*	
H5B	0.2058	0.5413	0.0142	0.051*	
H5C	0.2386	0.6539	0.0680	0.051*	
C6	0.4812 (3)	0.3553 (2)	0.23719 (12)	0.0294 (6)	
H6A	0.4361	0.2637	0.2235	0.035*	
H6B	0.5990	0.3686	0.2370	0.035*	
C7	0.4370 (3)	0.3868 (2)	0.29871 (12)	0.0284 (5)	
C8	0.5091 (3)	0.5012 (2)	0.33399 (12)	0.0305 (6)	
H8A	0.5904	0.5608	0.3193	0.037*	
C9	0.4627 (3)	0.5279 (2)	0.38995 (12)	0.0304 (6)	
H9A	0.5108	0.6066	0.4130	0.036*	
C10	0.3464 (3)	0.4409 (2)	0.41296 (12)	0.0276 (5)	
C11	0.2752 (3)	0.3270 (2)	0.37767 (12)	0.0279 (5)	
H11A	0.1957	0.2667	0.3927	0.033*	
C12	0.3182 (3)	0.3006 (2)	0.32147 (12)	0.0289 (6)	
H12A	0.2668	0.2233	0.2980	0.035*	
C13	0.2977 (3)	0.4734 (2)	0.47391 (12)	0.0292 (5)	
N14	0.2326 (3)	0.3702 (2)	0.49985 (10)	0.0290 (5)	
H14	0.250 (4)	0.298 (3)	0.4857 (13)	0.030 (8)*	
C15	0.1556 (3)	0.3663 (2)	0.55298 (12)	0.0276 (5)	
C16	0.0846 (3)	0.4640 (3)	0.57780 (12)	0.0314 (6)	
H16A	0.0945	0.5428	0.5620	0.038*	
C17	−0.0012 (3)	0.4419 (3)	0.62653 (13)	0.0339 (6)	
H17A	−0.0498	0.5080	0.6438	0.041*	
C18	−0.0199 (3)	0.3286 (3)	0.65133 (12)	0.0315 (6)	
C19	−0.1298 (4)	0.3073 (3)	0.70004 (14)	0.0437 (7)	
H19A	−0.0708	0.2871	0.7340	0.066*	
H19B	−0.2194	0.2357	0.6867	0.066*	
H19C	−0.1708	0.3851	0.7111	0.066*	
C20	0.0583 (3)	0.2345 (2)	0.62720 (11)	0.0264 (5)	
C21	0.1447 (3)	0.2542 (2)	0.57814 (12)	0.0274 (5)	
H21A	0.1971	0.1896	0.5617	0.033*	
N22	0.0369 (3)	0.1166 (2)	0.65091 (10)	0.0295 (5)	
H22	−0.053 (4)	0.098 (3)	0.6678 (15)	0.040 (9)*	
C23	0.1200 (3)	0.0202 (2)	0.64192 (11)	0.0264 (5)	
N24	0.0467 (3)	−0.0938 (2)	0.65697 (10)	0.0304 (5)	
C25	0.1278 (3)	−0.1869 (3)	0.64831 (13)	0.0341 (6)	
H25A	0.0816	−0.2684	0.6589	0.041*	
C26	0.2749 (3)	−0.1733 (2)	0.62498 (13)	0.0322 (6)	
H26A	0.3286	−0.2426	0.6189	0.039*	
C27	0.3399 (3)	−0.0522 (2)	0.61095 (11)	0.0267 (5)	
N28	0.2636 (3)	0.0452 (2)	0.62024 (9)	0.0270 (5)	
C29	0.4951 (3)	−0.0229 (2)	0.58435 (11)	0.0267 (5)	
C30	0.5691 (3)	−0.1204 (2)	0.56171 (12)	0.0290 (5)	
H30A	0.5167	−0.2065	0.5635	0.035*	
N31	0.7095 (3)	−0.1001 (2)	0.53756 (10)	0.0314 (5)	
C32	0.7788 (3)	0.0217 (3)	0.53364 (12)	0.0303 (6)	
H32A	0.8790	0.0380	0.5169	0.036*	
C33	0.7121 (3)	0.1247 (3)	0.55272 (12)	0.0308 (6)	
H33A	0.7635	0.2094	0.5479	0.037*	
C34	0.5695 (3)	0.1028 (2)	0.57896 (12)	0.0291 (5)	
H34A	0.5225	0.1724	0.5932	0.035*	
O1A	0.1518 (2)	0.22087 (16)	0.07622 (8)	0.0291 (4)	
O21A	0.3381 (2)	0.07732 (17)	0.12752 (9)	0.0346 (4)	
O22A	0.3131 (2)	−0.0904 (2)	0.06313 (10)	0.0396 (5)	
O41A	−0.2360 (2)	−0.33957 (17)	0.04481 (10)	0.0395 (5)	
O42A	−0.4305 (2)	−0.2407 (2)	0.03722 (12)	0.0493 (6)	
O61A	−0.3332 (3)	0.2150 (2)	0.07480 (12)	0.0520 (6)	
O62A	−0.1032 (3)	0.32666 (18)	0.06299 (11)	0.0427 (5)	
N2A	0.2595 (3)	−0.0043 (2)	0.08996 (10)	0.0287 (5)	
N4A	−0.2888 (3)	−0.2408 (2)	0.04636 (10)	0.0311 (5)	
N6A	−0.1906 (3)	0.2244 (2)	0.06879 (10)	0.0301 (5)	
C1A	0.0479 (3)	0.1211 (2)	0.07465 (10)	0.0242 (5)	
C2A	0.0909 (3)	−0.0022 (2)	0.07761 (11)	0.0253 (5)	
C3A	−0.0144 (3)	−0.1176 (2)	0.06785 (11)	0.0265 (5)	
H3AA	0.0223	−0.1956	0.0674	0.032*	
C4A	−0.1766 (3)	−0.1186 (2)	0.05852 (11)	0.0272 (5)	
C5A	−0.2330 (3)	−0.0062 (2)	0.06039 (11)	0.0261 (5)	
H5AA	−0.3445	−0.0084	0.0562	0.031*	
C6A	−0.1240 (3)	0.1095 (2)	0.06843 (11)	0.0259 (5)	
O1B	0.4787 (2)	0.68998 (16)	0.21876 (9)	0.0303 (4)	
O21B	0.2684 (2)	0.84672 (18)	0.19341 (9)	0.0348 (4)	
O22B	0.3412 (3)	0.9982 (2)	0.26585 (11)	0.0493 (6)	
O41B	0.8703 (3)	1.24447 (18)	0.21586 (10)	0.0441 (5)	
O42B	1.0597 (2)	1.13663 (18)	0.20751 (9)	0.0363 (5)	
O61B	0.9270 (2)	0.6835 (2)	0.16693 (9)	0.0392 (5)	
O62B	0.7440 (2)	0.58469 (18)	0.21578 (10)	0.0405 (5)	
N2B	0.3710 (3)	0.9196 (2)	0.22702 (11)	0.0316 (5)	
N4B	0.9186 (3)	1.1430 (2)	0.21198 (10)	0.0327 (5)	
N6B	0.8091 (3)	0.6809 (2)	0.19535 (10)	0.0285 (5)	
C1B	0.5803 (3)	0.7898 (2)	0.21517 (11)	0.0248 (5)	
C2B	0.5386 (3)	0.9143 (2)	0.22026 (11)	0.0267 (5)	
C3B	0.6441 (3)	1.0279 (2)	0.22022 (12)	0.0298 (6)	
H3BA	0.6099	1.1072	0.2255	0.036*	
C4B	0.8035 (3)	1.0239 (2)	0.21222 (11)	0.0281 (5)	
C5B	0.8561 (3)	0.9107 (2)	0.20437 (11)	0.0263 (5)	
H5BA	0.9650	0.9103	0.1982	0.032*	
C6B	0.7471 (3)	0.7972 (2)	0.20563 (11)	0.0257 (5)	

### Atomic displacement parameters (Å^2^)

**Table:**

	*U*^11^	*U*^22^	*U*^33^	*U*^12^	*U*^13^	*U*^23^
O1	0.0430 (11)	0.0197 (9)	0.0408 (11)	0.0000 (8)	0.0037 (9)	0.0007 (8)
N1	0.0236 (10)	0.0124 (10)	0.0347 (12)	−0.0010 (8)	0.0009 (8)	0.0036 (8)
N2	0.0293 (11)	0.0130 (10)	0.0334 (11)	0.0003 (8)	−0.0010 (9)	0.0033 (8)
C1	0.0243 (12)	0.0182 (12)	0.0379 (14)	0.0009 (9)	0.0048 (10)	0.0009 (10)
C2	0.0289 (13)	0.0207 (12)	0.0353 (14)	−0.0011 (10)	0.0052 (11)	0.0030 (10)
C3	0.0249 (12)	0.0173 (12)	0.0345 (13)	0.0006 (9)	−0.0003 (10)	0.0005 (9)
C4	0.0228 (12)	0.0179 (11)	0.0338 (13)	−0.0003 (9)	0.0027 (10)	0.0021 (9)
C5	0.0409 (16)	0.0195 (13)	0.0407 (15)	0.0019 (11)	−0.0042 (12)	0.0079 (11)
C6	0.0290 (13)	0.0189 (12)	0.0408 (15)	0.0051 (10)	−0.0005 (11)	0.0066 (10)
C7	0.0289 (13)	0.0214 (12)	0.0345 (14)	0.0037 (10)	−0.0034 (10)	0.0067 (10)
C8	0.0268 (13)	0.0197 (12)	0.0432 (15)	−0.0011 (10)	−0.0014 (11)	0.0087 (11)
C9	0.0315 (14)	0.0196 (12)	0.0361 (14)	−0.0020 (10)	−0.0058 (11)	0.0033 (10)
C10	0.0273 (13)	0.0183 (12)	0.0360 (14)	0.0027 (10)	−0.0041 (10)	0.0051 (10)
C11	0.0279 (13)	0.0176 (12)	0.0357 (14)	−0.0021 (10)	−0.0024 (10)	0.0070 (10)
C12	0.0310 (13)	0.0154 (11)	0.0373 (14)	−0.0012 (10)	−0.0064 (11)	0.0047 (10)
C13	0.0250 (12)	0.0241 (13)	0.0370 (14)	0.0040 (10)	−0.0023 (10)	0.0023 (10)
N14	0.0307 (12)	0.0198 (11)	0.0366 (12)	0.0066 (9)	0.0035 (9)	0.0011 (9)
C15	0.0225 (12)	0.0234 (12)	0.0342 (14)	0.0018 (10)	−0.0001 (10)	−0.0014 (10)
C16	0.0321 (14)	0.0206 (12)	0.0403 (15)	0.0038 (10)	0.0015 (11)	0.0026 (10)
C17	0.0354 (15)	0.0239 (13)	0.0427 (16)	0.0090 (11)	0.0077 (12)	−0.0017 (11)
C18	0.0299 (14)	0.0305 (14)	0.0329 (14)	0.0057 (11)	0.0032 (11)	−0.0001 (11)
C19	0.0502 (19)	0.0387 (17)	0.0472 (18)	0.0180 (14)	0.0163 (15)	0.0062 (13)
C20	0.0224 (12)	0.0215 (12)	0.0331 (13)	0.0004 (9)	−0.0005 (10)	0.0023 (10)
C21	0.0235 (12)	0.0221 (12)	0.0350 (14)	0.0036 (10)	0.0017 (10)	−0.0012 (10)
N22	0.0268 (11)	0.0266 (12)	0.0352 (12)	0.0034 (9)	0.0078 (9)	0.0056 (9)
C23	0.0272 (13)	0.0218 (12)	0.0278 (12)	0.0018 (10)	−0.0007 (10)	0.0004 (9)
N24	0.0291 (11)	0.0239 (11)	0.0349 (12)	−0.0015 (9)	0.0038 (9)	0.0017 (9)
C25	0.0356 (15)	0.0207 (13)	0.0420 (16)	−0.0040 (11)	0.0009 (12)	0.0055 (11)
C26	0.0339 (14)	0.0179 (12)	0.0440 (16)	0.0042 (10)	0.0013 (12)	0.0030 (11)
C27	0.0295 (13)	0.0216 (12)	0.0278 (13)	0.0046 (10)	−0.0018 (10)	0.0013 (9)
N28	0.0268 (11)	0.0213 (10)	0.0318 (11)	0.0029 (8)	0.0010 (9)	0.0026 (8)
C29	0.0270 (13)	0.0240 (13)	0.0285 (13)	0.0054 (10)	−0.0022 (10)	0.0024 (10)
C30	0.0319 (14)	0.0218 (12)	0.0333 (14)	0.0062 (10)	0.0002 (11)	0.0029 (10)
N31	0.0322 (12)	0.0281 (12)	0.0346 (12)	0.0100 (9)	0.0014 (9)	0.0011 (9)
C32	0.0258 (13)	0.0308 (14)	0.0335 (14)	0.0049 (11)	0.0008 (10)	0.0026 (11)
C33	0.0266 (13)	0.0245 (13)	0.0388 (15)	0.0014 (10)	−0.0021 (11)	0.0026 (11)
C34	0.0271 (13)	0.0217 (13)	0.0372 (14)	0.0052 (10)	−0.0009 (11)	−0.0003 (10)
O1A	0.0314 (10)	0.0166 (9)	0.0370 (10)	0.0004 (7)	0.0015 (8)	0.0026 (7)
O21A	0.0308 (10)	0.0213 (9)	0.0475 (12)	−0.0027 (8)	−0.0050 (8)	0.0048 (8)
O22A	0.0385 (11)	0.0330 (11)	0.0494 (12)	0.0142 (9)	0.0052 (9)	0.0018 (9)
O41A	0.0412 (11)	0.0168 (9)	0.0567 (13)	−0.0008 (8)	−0.0033 (9)	0.0037 (8)
O42A	0.0267 (11)	0.0258 (11)	0.0909 (18)	−0.0039 (8)	0.0026 (11)	0.0056 (11)
O61A	0.0363 (12)	0.0296 (11)	0.0924 (19)	0.0103 (9)	0.0199 (12)	0.0051 (11)
O62A	0.0376 (11)	0.0187 (10)	0.0708 (15)	0.0010 (8)	−0.0036 (10)	0.0125 (9)
N2A	0.0293 (11)	0.0201 (11)	0.0369 (12)	0.0033 (9)	0.0043 (9)	0.0073 (9)
N4A	0.0311 (12)	0.0205 (11)	0.0388 (13)	−0.0022 (9)	0.0040 (9)	0.0042 (9)
N6A	0.0331 (12)	0.0214 (11)	0.0345 (12)	0.0043 (9)	0.0013 (9)	0.0005 (9)
C1A	0.0299 (13)	0.0159 (11)	0.0250 (12)	0.0012 (10)	0.0022 (10)	0.0012 (9)
C2A	0.0272 (13)	0.0207 (12)	0.0275 (12)	0.0036 (10)	0.0026 (10)	0.0030 (9)
C3A	0.0318 (13)	0.0167 (11)	0.0304 (13)	0.0036 (10)	0.0037 (10)	0.0029 (9)
C4A	0.0324 (14)	0.0195 (12)	0.0268 (12)	−0.0014 (10)	0.0031 (10)	0.0024 (9)
C5A	0.0256 (12)	0.0223 (12)	0.0291 (13)	0.0016 (10)	0.0045 (10)	0.0029 (10)
C6A	0.0310 (13)	0.0196 (12)	0.0269 (12)	0.0043 (10)	0.0031 (10)	0.0032 (9)
O1B	0.0242 (9)	0.0149 (8)	0.0490 (11)	−0.0015 (7)	0.0018 (8)	0.0028 (7)
O21B	0.0261 (9)	0.0239 (10)	0.0516 (12)	−0.0020 (7)	−0.0013 (8)	0.0064 (8)
O22B	0.0426 (12)	0.0380 (12)	0.0659 (15)	0.0149 (10)	0.0069 (11)	−0.0120 (11)
O41B	0.0470 (13)	0.0176 (10)	0.0635 (14)	−0.0038 (9)	0.0048 (10)	0.0060 (9)
O42B	0.0337 (11)	0.0279 (10)	0.0409 (11)	−0.0099 (8)	0.0034 (8)	0.0049 (8)
O61B	0.0339 (11)	0.0346 (11)	0.0510 (12)	0.0102 (9)	0.0110 (9)	0.0052 (9)
O62B	0.0268 (10)	0.0191 (9)	0.0747 (15)	−0.0013 (8)	0.0050 (9)	0.0118 (9)
N2B	0.0314 (12)	0.0186 (11)	0.0450 (13)	0.0038 (9)	0.0050 (10)	0.0055 (9)
N4B	0.0387 (14)	0.0216 (11)	0.0326 (12)	−0.0061 (10)	0.0018 (10)	0.0033 (9)
N6B	0.0217 (10)	0.0215 (11)	0.0396 (12)	0.0005 (8)	−0.0022 (9)	0.0009 (9)
C1B	0.0266 (13)	0.0175 (12)	0.0278 (12)	0.0003 (10)	−0.0008 (10)	0.0008 (9)
C2B	0.0261 (13)	0.0200 (12)	0.0322 (13)	0.0020 (10)	0.0004 (10)	0.0010 (10)
C3B	0.0367 (14)	0.0166 (12)	0.0347 (14)	0.0035 (10)	−0.0008 (11)	0.0022 (10)
C4B	0.0323 (14)	0.0188 (12)	0.0289 (13)	−0.0047 (10)	0.0000 (10)	0.0036 (9)
C5B	0.0244 (12)	0.0241 (13)	0.0266 (12)	−0.0033 (10)	0.0002 (9)	0.0026 (9)
C6B	0.0267 (13)	0.0192 (12)	0.0289 (13)	0.0010 (10)	−0.0012 (10)	0.0015 (9)

### Geometric parameters (Å, °)

**Table:**

O1—C13	1.226 (3)	C23—N28	1.339 (3)
N1—C1	1.494 (3)	C23—N24	1.352 (3)
N1—C4	1.498 (3)	N24—C25	1.328 (4)
N1—C6	1.505 (3)	C25—C26	1.380 (4)
N1—H1	0.85 (3)	C25—H25A	0.9500
N2—C3	1.490 (3)	C26—C27	1.390 (4)
N2—C5	1.491 (3)	C26—H26A	0.9500
N2—C2	1.500 (3)	C27—N28	1.341 (3)
N2—H2	0.89 (4)	C27—C29	1.482 (4)
C1—C2	1.511 (4)	C29—C30	1.391 (4)
C1—H1A	0.9900	C29—C34	1.395 (4)
C1—H1B	0.9900	C30—N31	1.338 (4)
C2—H2A	0.9900	C30—H30A	0.9500
C2—H2B	0.9900	N31—C32	1.340 (4)
C3—C4	1.506 (3)	C32—C33	1.379 (4)
C3—H3A	0.9900	C32—H32A	0.9500
C3—H3B	0.9900	C33—C34	1.378 (4)
C4—H4A	0.9900	C33—H33A	0.9500
C4—H4B	0.9900	C34—H34A	0.9500
C5—H5A	0.9800	O1A—C1A	1.245 (3)
C5—H5B	0.9800	O21A—N2A	1.226 (3)
C5—H5C	0.9800	O22A—N2A	1.228 (3)
C6—C7	1.503 (4)	O41A—N4A	1.231 (3)
C6—H6A	0.9900	O42A—N4A	1.221 (3)
C6—H6B	0.9900	O61A—N6A	1.221 (3)
C7—C12	1.399 (4)	O62A—N6A	1.225 (3)
C7—C8	1.403 (4)	N2A—C2A	1.463 (3)
C8—C9	1.383 (4)	N4A—C4A	1.452 (3)
C8—H8A	0.9500	N6A—C6A	1.457 (3)
C9—C10	1.393 (4)	C1A—C2A	1.451 (3)
C9—H9A	0.9500	C1A—C6A	1.451 (4)
C10—C11	1.397 (3)	C2A—C3A	1.367 (3)
C10—C13	1.506 (4)	C3A—C4A	1.393 (4)
C11—C12	1.377 (4)	C3A—H3AA	0.9500
C11—H11A	0.9500	C4A—C5A	1.382 (4)
C12—H12A	0.9500	C5A—C6A	1.384 (3)
C13—N14	1.356 (3)	C5A—H5AA	0.9500
N14—C15	1.419 (3)	O1B—C1B	1.252 (3)
N14—H14	0.85 (3)	O21B—N2B	1.227 (3)
C15—C21	1.385 (4)	O22B—N2B	1.228 (3)
C15—C16	1.394 (4)	O41B—N4B	1.235 (3)
C16—C17	1.389 (4)	O42B—N4B	1.232 (3)
C16—H16A	0.9500	O61B—N6B	1.226 (3)
C17—C18	1.388 (4)	O62B—N6B	1.229 (3)
C17—H17A	0.9500	N2B—C2B	1.462 (3)
C18—C20	1.396 (4)	N4B—C4B	1.451 (3)
C18—C19	1.508 (4)	N6B—C6B	1.450 (3)
C19—H19A	0.9800	C1B—C6B	1.443 (4)
C19—H19B	0.9800	C1B—C2B	1.443 (3)
C19—H19C	0.9800	C2B—C3B	1.366 (4)
C20—C21	1.394 (4)	C3B—C4B	1.394 (4)
C20—N22	1.419 (3)	C3B—H3BA	0.9500
C21—H21A	0.9500	C4B—C5B	1.373 (4)
N22—C23	1.369 (3)	C5B—C6B	1.384 (3)
N22—H22	0.87 (4)	C5B—H5BA	0.9500
			
C1—N1—C4	108.6 (2)	C18—C20—N22	118.4 (2)
C1—N1—C6	111.1 (2)	C15—C21—C20	121.1 (2)
C4—N1—C6	111.62 (19)	C15—C21—H21A	119.5
C1—N1—H1	107 (2)	C20—C21—H21A	119.5
C4—N1—H1	109 (2)	C23—N22—C20	129.0 (2)
C6—N1—H1	109 (2)	C23—N22—H22	116 (2)
C3—N2—C5	110.9 (2)	C20—N22—H22	113 (2)
C3—N2—C2	110.4 (2)	N28—C23—N24	125.8 (2)
C5—N2—C2	110.8 (2)	N28—C23—N22	119.1 (2)
C3—N2—H2	105 (2)	N24—C23—N22	115.2 (2)
C5—N2—H2	110 (2)	C25—N24—C23	115.0 (2)
C2—N2—H2	109 (2)	N24—C25—C26	124.4 (2)
N1—C1—C2	110.9 (2)	N24—C25—H25A	117.8
N1—C1—H1A	109.5	C26—C25—H25A	117.8
C2—C1—H1A	109.5	C25—C26—C27	116.1 (2)
N1—C1—H1B	109.5	C25—C26—H26A	121.9
C2—C1—H1B	109.5	C27—C26—H26A	121.9
H1A—C1—H1B	108.0	N28—C27—C26	121.4 (2)
N2—C2—C1	112.1 (2)	N28—C27—C29	116.0 (2)
N2—C2—H2A	109.2	C26—C27—C29	122.6 (2)
C1—C2—H2A	109.2	C23—N28—C27	117.3 (2)
N2—C2—H2B	109.2	C30—C29—C34	117.4 (2)
C1—C2—H2B	109.2	C30—C29—C27	121.1 (2)
H2A—C2—H2B	107.9	C34—C29—C27	121.5 (2)
N2—C3—C4	111.5 (2)	N31—C30—C29	123.9 (2)
N2—C3—H3A	109.3	N31—C30—H30A	118.0
C4—C3—H3A	109.3	C29—C30—H30A	118.0
N2—C3—H3B	109.3	C30—N31—C32	117.1 (2)
C4—C3—H3B	109.3	N31—C32—C33	123.3 (3)
H3A—C3—H3B	108.0	N31—C32—H32A	118.4
N1—C4—C3	111.2 (2)	C33—C32—H32A	118.4
N1—C4—H4A	109.4	C34—C33—C32	119.0 (2)
C3—C4—H4A	109.4	C34—C33—H33A	120.5
N1—C4—H4B	109.4	C32—C33—H33A	120.5
C3—C4—H4B	109.4	C33—C34—C29	119.2 (2)
H4A—C4—H4B	108.0	C33—C34—H34A	120.4
N2—C5—H5A	109.5	C29—C34—H34A	120.4
N2—C5—H5B	109.5	O21A—N2A—O22A	123.7 (2)
H5A—C5—H5B	109.5	O21A—N2A—C2A	118.4 (2)
N2—C5—H5C	109.5	O22A—N2A—C2A	117.9 (2)
H5A—C5—H5C	109.5	O42A—N4A—O41A	123.1 (2)
H5B—C5—H5C	109.5	O42A—N4A—C4A	118.6 (2)
C7—C6—N1	112.6 (2)	O41A—N4A—C4A	118.2 (2)
C7—C6—H6A	109.1	O61A—N6A—O62A	122.0 (2)
N1—C6—H6A	109.1	O61A—N6A—C6A	118.4 (2)
C7—C6—H6B	109.1	O62A—N6A—C6A	119.6 (2)
N1—C6—H6B	109.1	O1A—C1A—C2A	121.3 (2)
H6A—C6—H6B	107.8	O1A—C1A—C6A	126.9 (2)
C12—C7—C8	118.6 (3)	C2A—C1A—C6A	111.7 (2)
C12—C7—C6	119.2 (2)	C3A—C2A—C1A	124.6 (2)
C8—C7—C6	122.2 (2)	C3A—C2A—N2A	117.3 (2)
C9—C8—C7	120.4 (2)	C1A—C2A—N2A	118.0 (2)
C9—C8—H8A	119.8	C2A—C3A—C4A	118.7 (2)
C7—C8—H8A	119.8	C2A—C3A—H3AA	120.6
C8—C9—C10	120.9 (2)	C4A—C3A—H3AA	120.6
C8—C9—H9A	119.5	C5A—C4A—C3A	121.5 (2)
C10—C9—H9A	119.5	C5A—C4A—N4A	119.4 (2)
C9—C10—C11	118.4 (3)	C3A—C4A—N4A	119.1 (2)
C9—C10—C13	119.6 (2)	C4A—C5A—C6A	118.8 (2)
C11—C10—C13	122.1 (2)	C4A—C5A—H5AA	120.6
C12—C11—C10	121.2 (2)	C6A—C5A—H5AA	120.6
C12—C11—H11A	119.4	C5A—C6A—C1A	124.0 (2)
C10—C11—H11A	119.4	C5A—C6A—N6A	116.2 (2)
C11—C12—C7	120.4 (2)	C1A—C6A—N6A	119.7 (2)
C11—C12—H12A	119.8	O21B—N2B—O22B	123.7 (2)
C7—C12—H12A	119.8	O21B—N2B—C2B	118.5 (2)
O1—C13—N14	123.9 (3)	O22B—N2B—C2B	117.8 (2)
O1—C13—C10	121.8 (2)	O42B—N4B—O41B	123.7 (2)
N14—C13—C10	114.3 (2)	O42B—N4B—C4B	117.7 (2)
C13—N14—C15	129.0 (2)	O41B—N4B—C4B	118.7 (2)
C13—N14—H14	117 (2)	O61B—N6B—O62B	122.5 (2)
C15—N14—H14	113 (2)	O61B—N6B—C6B	118.1 (2)
C21—C15—C16	120.2 (2)	O62B—N6B—C6B	119.4 (2)
C21—C15—N14	116.1 (2)	O1B—C1B—C6B	126.3 (2)
C16—C15—N14	123.6 (2)	O1B—C1B—C2B	121.7 (2)
C17—C16—C15	117.5 (2)	C6B—C1B—C2B	112.0 (2)
C17—C16—H16A	121.2	C3B—C2B—C1B	125.1 (2)
C15—C16—H16A	121.2	C3B—C2B—N2B	117.4 (2)
C18—C17—C16	123.6 (2)	C1B—C2B—N2B	117.6 (2)
C18—C17—H17A	118.2	C2B—C3B—C4B	117.9 (2)
C16—C17—H17A	118.2	C2B—C3B—H3BA	121.1
C17—C18—C20	117.6 (3)	C4B—C3B—H3BA	121.1
C17—C18—C19	120.0 (3)	C5B—C4B—C3B	122.3 (2)
C20—C18—C19	122.2 (3)	C5B—C4B—N4B	118.5 (2)
C18—C19—H19A	109.5	C3B—C4B—N4B	119.3 (2)
C18—C19—H19B	109.5	C4B—C5B—C6B	118.6 (2)
H19A—C19—H19B	109.5	C4B—C5B—H5BA	120.7
C18—C19—H19C	109.5	C6B—C5B—H5BA	120.7
H19A—C19—H19C	109.5	C5B—C6B—C1B	124.1 (2)
H19B—C19—H19C	109.5	C5B—C6B—N6B	115.8 (2)
C21—C20—C18	119.8 (2)	C1B—C6B—N6B	120.1 (2)
C21—C20—N22	121.6 (2)		
			
C4—N1—C1—C2	58.2 (2)	C27—C29—C30—N31	−179.5 (2)
C6—N1—C1—C2	−178.6 (2)	C29—C30—N31—C32	−2.2 (4)
C3—N2—C2—C1	53.6 (3)	C30—N31—C32—C33	−0.4 (4)
C5—N2—C2—C1	176.8 (2)	N31—C32—C33—C34	2.2 (4)
N1—C1—C2—N2	−56.6 (3)	C32—C33—C34—C29	−1.4 (4)
C5—N2—C3—C4	−177.2 (2)	C30—C29—C34—C33	−1.0 (4)
C2—N2—C3—C4	−54.0 (3)	C27—C29—C34—C33	−178.6 (2)
C1—N1—C4—C3	−59.2 (3)	O1A—C1A—C2A—C3A	−170.2 (2)
C6—N1—C4—C3	177.9 (2)	C6A—C1A—C2A—C3A	8.4 (4)
N2—C3—C4—N1	58.1 (3)	O1A—C1A—C2A—N2A	7.6 (4)
C1—N1—C6—C7	−179.7 (2)	C6A—C1A—C2A—N2A	−173.8 (2)
C4—N1—C6—C7	−58.3 (3)	O21A—N2A—C2A—C3A	−140.1 (2)
N1—C6—C7—C12	107.2 (3)	O22A—N2A—C2A—C3A	37.9 (3)
N1—C6—C7—C8	−71.7 (3)	O21A—N2A—C2A—C1A	41.9 (3)
C12—C7—C8—C9	−0.3 (4)	O22A—N2A—C2A—C1A	−140.1 (2)
C6—C7—C8—C9	178.6 (2)	C1A—C2A—C3A—C4A	−4.9 (4)
C7—C8—C9—C10	1.5 (4)	N2A—C2A—C3A—C4A	177.2 (2)
C8—C9—C10—C11	−1.3 (4)	C2A—C3A—C4A—C5A	−1.6 (4)
C8—C9—C10—C13	−179.5 (2)	C2A—C3A—C4A—N4A	178.2 (2)
C9—C10—C11—C12	0.0 (4)	O42A—N4A—C4A—C5A	1.1 (4)
C13—C10—C11—C12	178.1 (2)	O41A—N4A—C4A—C5A	179.9 (2)
C10—C11—C12—C7	1.2 (4)	O42A—N4A—C4A—C3A	−178.7 (3)
C8—C7—C12—C11	−1.0 (4)	O41A—N4A—C4A—C3A	0.1 (4)
C6—C7—C12—C11	−180.0 (2)	C3A—C4A—C5A—C6A	3.7 (4)
C9—C10—C13—O1	23.7 (4)	N4A—C4A—C5A—C6A	−176.1 (2)
C11—C10—C13—O1	−154.5 (3)	C4A—C5A—C6A—C1A	0.5 (4)
C9—C10—C13—N14	−156.9 (2)	C4A—C5A—C6A—N6A	178.8 (2)
C11—C10—C13—N14	25.0 (4)	O1A—C1A—C6A—C5A	172.4 (2)
O1—C13—N14—C15	8.6 (4)	C2A—C1A—C6A—C5A	−6.1 (3)
C10—C13—N14—C15	−170.8 (2)	O1A—C1A—C6A—N6A	−5.8 (4)
C13—N14—C15—C21	−160.7 (3)	C2A—C1A—C6A—N6A	175.8 (2)
C13—N14—C15—C16	23.3 (4)	O61A—N6A—C6A—C5A	15.5 (4)
C21—C15—C16—C17	−2.5 (4)	O62A—N6A—C6A—C5A	−164.3 (2)
N14—C15—C16—C17	173.3 (3)	O61A—N6A—C6A—C1A	−166.2 (3)
C15—C16—C17—C18	−0.2 (4)	O62A—N6A—C6A—C1A	14.0 (4)
C16—C17—C18—C20	2.9 (4)	O1B—C1B—C2B—C3B	176.0 (3)
C16—C17—C18—C19	−173.3 (3)	C6B—C1B—C2B—C3B	−4.2 (4)
C17—C18—C20—C21	−3.0 (4)	O1B—C1B—C2B—N2B	−2.9 (4)
C19—C18—C20—C21	173.1 (3)	C6B—C1B—C2B—N2B	176.9 (2)
C17—C18—C20—N22	−178.2 (2)	O21B—N2B—C2B—C3B	132.4 (3)
C19—C18—C20—N22	−2.1 (4)	O22B—N2B—C2B—C3B	−46.8 (4)
C16—C15—C21—C20	2.4 (4)	O21B—N2B—C2B—C1B	−48.7 (3)
N14—C15—C21—C20	−173.7 (2)	O22B—N2B—C2B—C1B	132.2 (3)
C18—C20—C21—C15	0.4 (4)	C1B—C2B—C3B—C4B	2.8 (4)
N22—C20—C21—C15	175.5 (2)	N2B—C2B—C3B—C4B	−178.3 (2)
C21—C20—N22—C23	17.2 (4)	C2B—C3B—C4B—C5B	0.2 (4)
C18—C20—N22—C23	−167.7 (3)	C2B—C3B—C4B—N4B	−180.0 (2)
C20—N22—C23—N28	18.2 (4)	O42B—N4B—C4B—C5B	−3.4 (4)
C20—N22—C23—N24	−162.5 (2)	O41B—N4B—C4B—C5B	176.1 (2)
N28—C23—N24—C25	−0.6 (4)	O42B—N4B—C4B—C3B	176.7 (2)
N22—C23—N24—C25	−179.8 (2)	O41B—N4B—C4B—C3B	−3.7 (4)
C23—N24—C25—C26	−1.0 (4)	C3B—C4B—C5B—C6B	−1.3 (4)
N24—C25—C26—C27	1.0 (4)	N4B—C4B—C5B—C6B	178.9 (2)
C25—C26—C27—N28	0.6 (4)	C4B—C5B—C6B—C1B	−0.5 (4)
C25—C26—C27—C29	−178.6 (2)	C4B—C5B—C6B—N6B	178.3 (2)
N24—C23—N28—C27	2.1 (4)	O1B—C1B—C6B—C5B	−177.2 (3)
N22—C23—N28—C27	−178.7 (2)	C2B—C1B—C6B—C5B	3.0 (4)
C26—C27—N28—C23	−2.0 (4)	O1B—C1B—C6B—N6B	4.1 (4)
C29—C27—N28—C23	177.2 (2)	C2B—C1B—C6B—N6B	−175.7 (2)
N28—C27—C29—C30	−166.7 (2)	O61B—N6B—C6B—C5B	−25.5 (3)
C26—C27—C29—C30	12.6 (4)	O62B—N6B—C6B—C5B	153.6 (2)
N28—C27—C29—C34	10.8 (4)	O61B—N6B—C6B—C1B	153.3 (2)
C26—C27—C29—C34	−170.0 (3)	O62B—N6B—C6B—C1B	−27.6 (4)
C34—C29—C30—N31	3.0 (4)		

### Hydrogen-bond geometry (Å, °)

**Table:**

*D*—H···*A*	*D*—H	H···*A*	*D*···*A*	*D*—H···*A*
N1—H1···O1B	0.85 (3)	1.85 (3)	2.658 (3)	157 (3)
N1—H1···O62B	0.85 (3)	2.35 (3)	2.890 (3)	122 (2)
N2—H2···O1A	0.89 (4)	1.85 (4)	2.678 (3)	154 (3)
N2—H2···O62A	0.89 (4)	2.41 (4)	3.009 (3)	125 (3)
N14—H14···N31^i^	0.85 (3)	2.23 (3)	3.069 (3)	171 (3)
C5—H5B···O41A^ii^	0.98	2.48	3.258 (4)	136
C4—H4B···O42B^iii^	0.99	2.33	3.199 (3)	146
C3—H3A···O61B^iv^	0.99	2.57	3.199 (3)	121
C3—H3B···O1B	0.99	2.34	3.072 (3)	130
C12—H12A···O42B^iii^	0.95	2.63	3.423 (3)	142
C19—H19A···O61B^v^	0.98	2.50	3.435 (4)	159
C19—H19A···N6B^v^	0.98	2.65	3.541 (4)	152

Symmetry codes: (i) −*x*+1, −*y*, −*z*+1; (ii) −*x*, −*y*, −*z*; (iii) *x*−1, *y*−1, *z*; (iv) *x*−1, *y*, *z*; (v) −*x*+1, −*y*+1, −*z*+1.

### Table 2 π-Ring hydrogen-bond geometry (Å, °) for (I).

*Cg*5 is the centroid of the C15–C21 ring.

**Table:**

*D*—H···*A*	*D*—H	H···*A*	*D*···*A*	*D*—H···*A*
C33—H33*A*···*Cg*5^vi^	0.95	2.90	3.545 (8)	127

Symmetry code: (vi) x+1, y, z.

### Table 3 π–π stacking geometry (Å, °) for (I).

**Table:**

*Cg*2···*Cg*7^v^	3.740 (4)
*Cg*3···*Cg*3^v^	3.496 (7)
*Cg*6···*Cg*6^vii^	3.396 (0)

Symmetry codes: (v) -x+1, -y+1, -z+1; (vii) -x+2, -y+2, -z. Notes: *Cg*2, *Cg*3, *Cg*6 and *Cg*7 are the centroids of the C25/C26/C27/N28/C23/N4, C32/C33/C34/C29//C30/N31, C1A–C6A and C1B–C6B rings, respectively.
